# Lung Adenocarcinoma Cells Promote Self-Migration and Self-Invasion by Activating Neutrophils to Upregulate Notch3 Expression of Cancer Cells

**DOI:** 10.3389/fmolb.2021.762729

**Published:** 2022-01-18

**Authors:** Weidong Peng, Youjing Sheng, Han Xiao, Yuanzi Ye, Louis Boafo Kwantwi, Lanqing Cheng, Yuanchong Wang, Jiegou Xu, Qiang Wu

**Affiliations:** ^1^ Department of Pathology, The First Affiliated Hospital of Anhui Medical University, Hefei, China; ^2^ Department of Pathology, School of Basic Medical Science, Anhui Medical University, Hefei, China; ^3^ Department of Pathology, The Second Affiliated Hospital of Anhui Medical University, Hefei, China; ^4^ Department of Neonatology, Anhui Provincial Children’s Hospital, Hefei, China; ^5^ Department of Immunology, School of Basic Medical Science, Anhui Medical University, Hefei, China

**Keywords:** lung adenocarcinoma, Notch3, migration, invasion, tumor-associated neutrophils

## Abstract

**Background:** Invasion and migration of cancer cells play a key role in lung cancer progression and metastasis. Tumor-associated neutrophils (TANs) are related to poor prognosis in many types of cancer. However, the role of TANs in lung cancer is controversial. In this study, we investigated the effect of TANs on the invasion and migration of lung adenocarcinoma.

**Methods: **Immunohistochemistry was performed to detect the density of infiltrating TANs and the expression of Notch3 in 100 lung adenocarcinoma tissues. Flow cytometry was used to observe the viability of neutrophils, which were isolated from healthy peripheral blood and then exposed to the supernatant of cultured lung adenocarcinoma cell lines. After treating with tumor-associated neutrophils culture supernatant, NeuCS (supernatant of cultured neutrophils), tumor cells culture supernatant, Medium (serum-free medium), respectively, the migration and invasion of the lung cancer cells before and after transfected by si-Notch3 were detected by transwell assay and wound healing assay. Kaplan-Meier plotter (http://kmplot.com/analysis/index.php?p) was used to analyze the prognostic role of the density of TANs on lung adenocarcinoma and TIMER ((http://cistrome.dfci.harvard.edu/TIMER/) was used to detect the expression of Notch3 on lung adenocarcinoma.

**Results:** The infiltration of TANs was observed in the parenchyma and stroma of the lung adenocarcinoma, the density of TANs was positively related to the TNM stage and negatively related to the differentiation and prognosis. Notch3 expression of cancer cells was negatively related to the tumor differentiation and prognosis. Compared to quiescent neutrophils, the viability of TCCS-activated neutrophils was enhanced. Both migration and invasion of A549 and PC9 cells were significantly promoted by TANs, while after knocking down Notch3, the migration and invasion of the cancer cells were not affected by TANs. Bioinformatics analysis showed that the density of TANs and the expression of Notch3 were related to the poor prognosis.

**Conclusion:** The results indicated that lung adenocarcinoma cells promote self-invasion and self-migration by activating neutrophils to upregulate the Notch3 expression of cancer cells. The density of infiltrating TANs may be a novel marker for the poor prognosis of lung adenocarcinoma. Targeting TANs might be a potential therapeutic strategy for lung cancer treatment.

## Introduction

Lung cancer continues to be the most commonly diagnosed cancer type and the leading cause of cancer-related death worldwide (Bade and Dela, 2020). Although the overall survival has improved with surgical advances, the 5-years survival rate is still less than 17% (Hirsch et al., 2017). This poor prognostic outcome is primarily attributed to recurrence and metastasis existing in postoperative (Yan et al., 2017). Therefore, exploring further possible prognostic and predictive biomarkers and a potential therapeutic strategy for lung cancer treatment is necessary.

Current evidence has indicated that cross-talk between tumor cells and immune cells creates a unique microenvironment that promotes tumor cell growth, invasion, and metastasis (Zhou et al., 2016). As the most abundant immune cells in the microenvironment, Neutrophils respond to various inflammatory and cancerous signals. Emerging evidence has suggested that tumor-associated neutrophils (TANs) are involved in the malignant progression of several cancer types (Masucci et al., 2019). The infiltration of neutrophils in tumors has been associated with a poor prognosis. In renal cell carcinoma, the infiltration of neutrophils has been shown to correlate with short recurrence-free survival (RFS) and overall survival (OS) in patients (Jensen et al., 2009). Wang et al. have indicated that increased intratumoral neutrophils independently predict poor prognostic outcomes in esophageal carcinoma (Wang et al., 2014). However, the role of TANs in lung cancer has been a controversial issue. Carus et al. reported the density of CD66b + neutrophils has no significant correlation with RFS or OS in non-small cell lung cancer (Carus et al., 2013), While Rakaee et al. noted that the density of CD66b + TANs to be an independent negative prognostic factor in patients with lung adenocarcinoma (Rakaee et al., 2016). These contradictory observations highlight the diversity of TANs and a pressing need to further evaluate their roles and underlying mechanisms in lung cancer.

Notch signaling is crucial for cell fate and normal embryonic development (Liang et al., 2019). However, recent evidence showed that the Notch family plays a vital role in malignancies (Garcia and Kandel, 2012). Among the four receptors (Notch1-4) in the Notch family, accumulated evidence has shown the overexpression of Notch3 to be associated with recurrence and metastasis in some cancer types (Kim et al., 2017; Tang et al., 2019)). In lung cancer, Notch3 has been reported to be associated with poor prognosis (Ye et al., 2013). In gastric cancer, the expression of Notch3 is correlated with the infiltration of immune cells, including CD8^+^ T cells, Tregs, and M2 macrophages (Cui et al., 2020). According to Yan et al., Notch3 expression is closely related to the infiltration of M2 macrophage in melanoma tissues (Yan et al., 2021). Although the aforementioned evidence suggests an interaction between Notch3 and immune cells, how Notch3 interacts with immune cells, particularly TANs in lung cancer, remained unexplored.

In this study, we investigated the prognostic role of CD66b + TANs in lung adenocarcinoma tissues and found a correlation between the density of TANs and the expression of Notch3. We further investigated the activation of neutrophils by supernatant of cultured lung adenocarcinoma cells and the effect of these TANs on the migration and invasion of lung cancer cells via the Notch3 pathway.

## Materials and Methods

### Patients and Specimens

Formalin-fixed-paraffin-embedded (FFPE) tissue specimens were obtained from 100 lung adenocarcinoma patients who underwent surgical operation from 2014 to 2017 at the First Affiliated Hospital of Anhui Medical University. Patients included in this study had not received any preoperative adjuvant therapy. All specimens and protocols used in this study were approved by the Ethics Committee of Anhui Medical University (No. 2021H004). The protocols used to be under the ethical standards of the Biomedical Ethics Committee of Anhui Medical University Committee and the 1964 Helsinki Declaration.

### Immunohistochemistry

FFPE samples sliced into 4 μm sections were deparaffinized in xylene and graded alcohol, followed by hydration. Antigen retrieval was performed in a pressure cooker containing 0.01 M sodium citrate buffer (pH 6.0) for 10 min. Tissue sections were incubated with either anti-CD66b (Cat.No.555723, BD Pharmingen) or anti-Notch3 (Cat.No.5276S, Cell Signaling Technology) overnight at 4°C. After blocking by endogenous peroxidase for 30 min, samples were incubated with the HRP-labeled sheep anti-mouse/rabbit IgG polymer (Cat.No.PV-6000, ZSGB-BIO) for 20 min at room temperature. Finally, 3,3′-Diaminobenzidine (DAB) was used as chromogen, and the sections were counterstained with hematoxylin.

### Scoring Stained Sections

The density of CD66b + neutrophils infiltrating in tumor parenchyma was evaluated as previously reported (Wang et al., 2019). Briefly, CD66b + neutrophils were counted under ten random microscopic high power fields (×40 objective lenses). According to the median value of all samples, CD66b + neutrophils were divided into high-density and low-density groups. Notch3 immunostaining intensity was graded on 0–3 scales: 0, absence of staining; 1, weak staining; 2, moderate staining; 3, strong staining. Notch3 positive cancer cells were similarly scored on a scale of 0–4 as follows: 0, 0–5% positive tumor cells; 1, 6–25% positive tumor cells; 2, 26–50% positive tumor cells; 3, 51–75% positive tumor cells; 4, >75% tumor cells. The IHC scores (0–12) were obtained by multiplying the staining intensity by the percentage scores; the score <4 was negative, ≥4 was positive.

### Isolation and Culture of Neutrophils

Blood obtained from healthy adults was centrifuged at 250 g for 15–20 min. After discarding the plasma, normal saline was added. The diluted blood was carefully added onto a Ficoll solution and centrifuged at 450 g for 25 min. After discarding the supernatant, RBC lysing buffer was added twice each for 10 min. After centrifuging at 1500 rpm and discarding the supernatant, the cells were washed twice with PBS, and the cells were resuspended in a serum-free medium.

### Preparation of Tumor Cells Culture Supernatant and NeuCS and Tumor-Associated Neutrophils Culture Supernatant

TCCS (tumor cells culture supernatant) was obtained from the supernatant of A549 and PC-9 lung cancer cells cultured for 48 h in a serum-free medium. NeuCS (Neutrophils culture supernatant) was obtained from the supernatant of neutrophils cultured in the serum-free medium for 10 h. TANCS (tumor-associated neutrophils culture supernatant) was obtained from the supernatant of neutrophils stimulated by TCCS for 10 h.

### Flowcytometry

The viability of TANs and neutrophils was determined by Annexin-V/PI apoptosis detection kits (Biobest). The data were analyzed by FlowJo (Version. 7.6.1).

### Migration and Invasion Assay

Migration and invasion were performed in an 8 μm 24-well culture insert (Corning Company). For invasion assay, Matrigel (BD Company) diluted with the serum-free medium was precoated in the chambers and solidified for 30 min at 37°C. Lung cancer cells were added to the upper chamber, whereas TANCS, NeuCS, TCCS, and medium were added to the lower chamber, respectively. The cancer cells were cultured for 7 and 12 h for migration and invasion assay, respectively. *Cancer* cells attached to the underside of the chambers were fixed and stained. Quantification was performed by evaluating the mean number of cells in five microscopic fields per chamber.

### Wound Healing Assay

The cells were seeded in a 6-well plate at 3.6 × 105 cells per well and were cultured overnight. After the next day, 75 pmol si-Notch3 or negative control was transfected by using Lipofectamine3000. The transfected cells and untransfected cells were cultured in opti-mem. After 6 h, each group of cells was treated with TANCS, NeuCS, TCCS, and medium, respectively. At the same time, the cells were grown to confluence and wounded by dragging a 0.2-ml pipette tip through the monolayer. The extent of cell migration was observed. The rate of wound closure was expressed as a percentage of the initial scraped gap.

### Real-Time Polymerase Chain Reaction

RNA was extracted from A549 and PC9 cells using TRIzol reagent (ThermoFisher Scientific), and cDNA was made by reverse transcription with (Evo M-MLV RT Master Mix) according to the manufacturer’s instructions. qRT-PCR was performed using (SYBR^®^ Green Pro Taq HS Premix) in 20 μL reactions. Primers were designed using Primer3 software and purchased from Sangon Biotech: GAPDH (Sangon Biotech, B662104-0001), Notch3 Fw 5′-GCT​ACA​CTG​GAC​CTC​GCT​GT-3′, Notch3 Rv 5′-AGA​CCC​CAC​CGT​TGA​CAC​AG-3’. Reactions were carried out in LightCycler^®^ 96 Application Software (Roche). The cycling program used was 95°C for 30 s, followed by 40 cycles of 95°C for 5 s, 60°C for 30 s. Data were analyzed using GAPDH as a reference gene.

### Western Blotting

Proteins extracted using RIPA buffer were loaded onto SDS–polyacrylamide gels and then transferred to PVDF membranes. Membranes were blocked with 5% nonfat milk in TBS-T for 1 h at room temperature. Membranes were incubated overnight at 4°C with the following primary antibodies: NOTCH3 (Cat.No. 5276S, CellSignaling Technology) and GAPDH (Cat.No. abs132004, Absin). After incubating the membranes with peroxidase-conjugated secondary antibodies (Cat.No. ZB-2301, ZSGB-BIO) for 2 h, the reaction was visualized by an enhanced chemiluminescence assay.

### Bioinformatics Analysis

The Kaplan–Meier plotter ofers a means of readily exploring the impact of a wide array of genes on patient survival in 21 diferent types of cancer, with large sample sizes for the lung adenocarcionma (*n* = 865) cohorts. We therefore used this database to explore the association between Notch3 expression and outcome in patients with lung cancers (http://kmplot. com/analysis/).

TIMER (https://cistrome.shinyapps.io/timer/) is a database designed for the analysis of immune cell infltrates in multiple cancers. This database employs pathological examination-validated statistical methodology to estimate infltration by neutrophils, macrophages, dendritic cells, B cells and CD4+/CD8+ T cells into tumors. We therefore explore the efect of neutrophils infltration on the survival rate of lung adenocarcinoma patients (*n* = 496).

### Statistics

All experiments were carried out three times. The data were expressed as a mean ± standard deviation (SD). Comparison between groups was analyzed by chi-square test. Kaplan-meier was used for survival analysis and *p*-value < 0.05 was considered statistically significant.

## Results

### The Infiltrating of TANs and the Expression of Notch3 in Lung Adenocarcinoma

**FIGURE 1:**
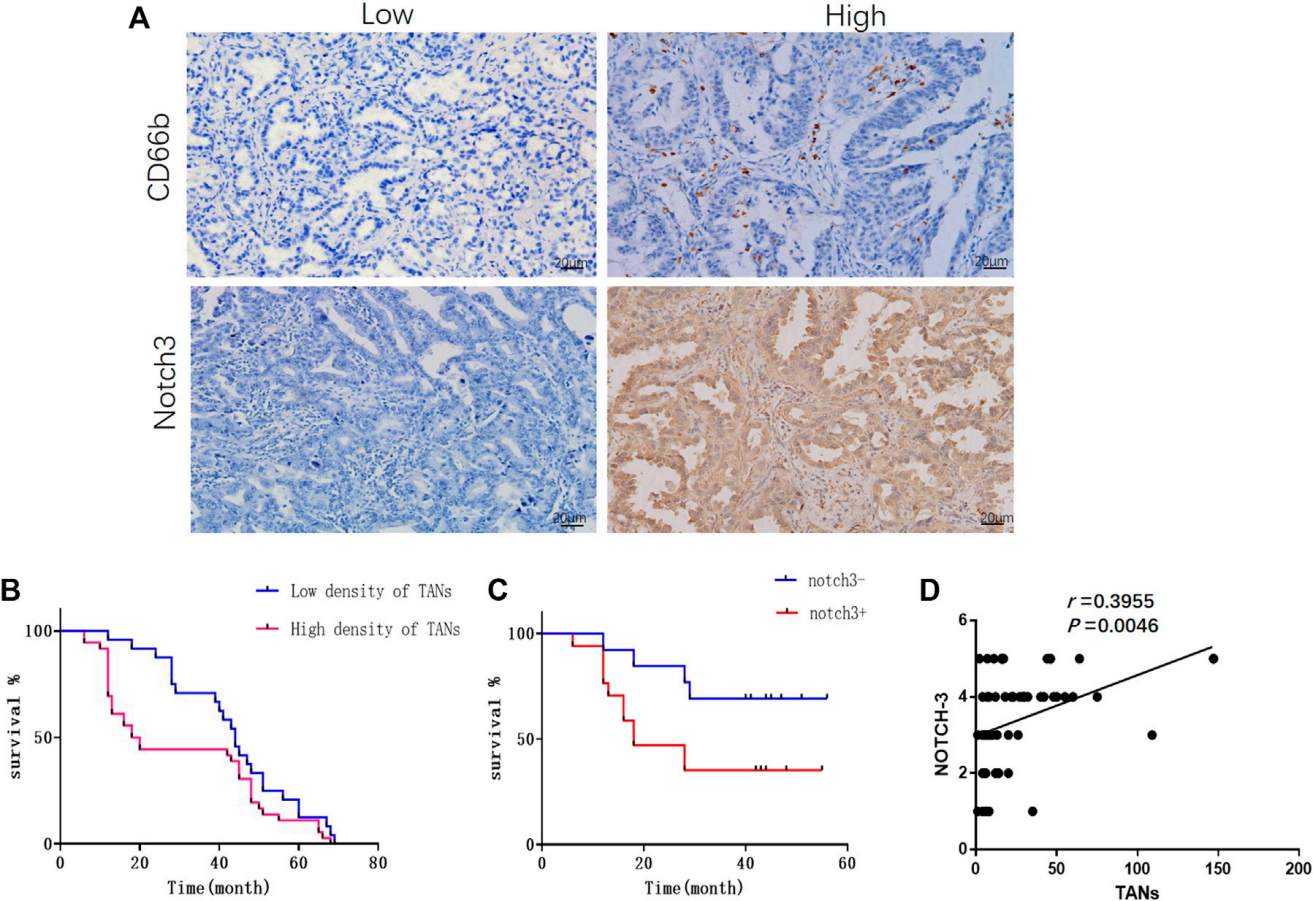
The infiltration of TANs and the expression of Notch3 in lung adenocarcinoma were associated with poor prognosis. **(A)** Representative images for immunohistochemical staining of CD66b and Notch3 in lung adenocarcinoma. **(B)** Survival analysis showed that the density of TANs was negatively correlated with disease-free survival (DFS). **(C)** Survival analysis showed that the expression of Notch3 was negatively correlated to DFS. **(D)** The correlation between the density of TANs and the expression of Notch3 in lung adenocarcinoma.

TANs were observed in both parenchyma and stroma of lung adenocarcinoma (Figure 1A). Only those infiltrating in tumor parenchyma were counted. The density of TANs infiltrating in solid and micropapillary adenocarcinoma was significantly higher than that in lepidic adenocarcinoma. The density of TANs showed a positive correlation to the TNM stage but correlated negatively to tumor differentiation (*p* < 0.05, Supplementary Table S1). Survival analysis showed that the density of TANs was negatively associated with disease-free survival (DFS) (*p* < 0.05, Figure 1B).

Notch3 was mainly expressed in the cytoplasm of cancer cells as detected by immunohistochemistry (Figure 1A), and the expression of Notch3 in solid adenocarcinoma was significantly higher than that in lepidic adenocarcinoma. The expression of Notch3 was negatively correlated to tumor differentiation (*p* < 0.05), but showed no significant correlation with gender, age, tumor size, TNM stage, or lymph node metastasis (Supplementary Table S1). Survival analysis showed that the expression of Notch3 was negatively correlated to DFS (*p* < 0.05, Figure 1C). In lung adenocarcinoma tissues, the infiltration of TANS was positively correlated with the expression of Notch3 (*p* < 0.05, Figure 1D).

### Neutrophils Were Activated to TANs by Supernatant of Cultured Lung Adenocarcinoma Cell Lines

**FIGURE 2:**
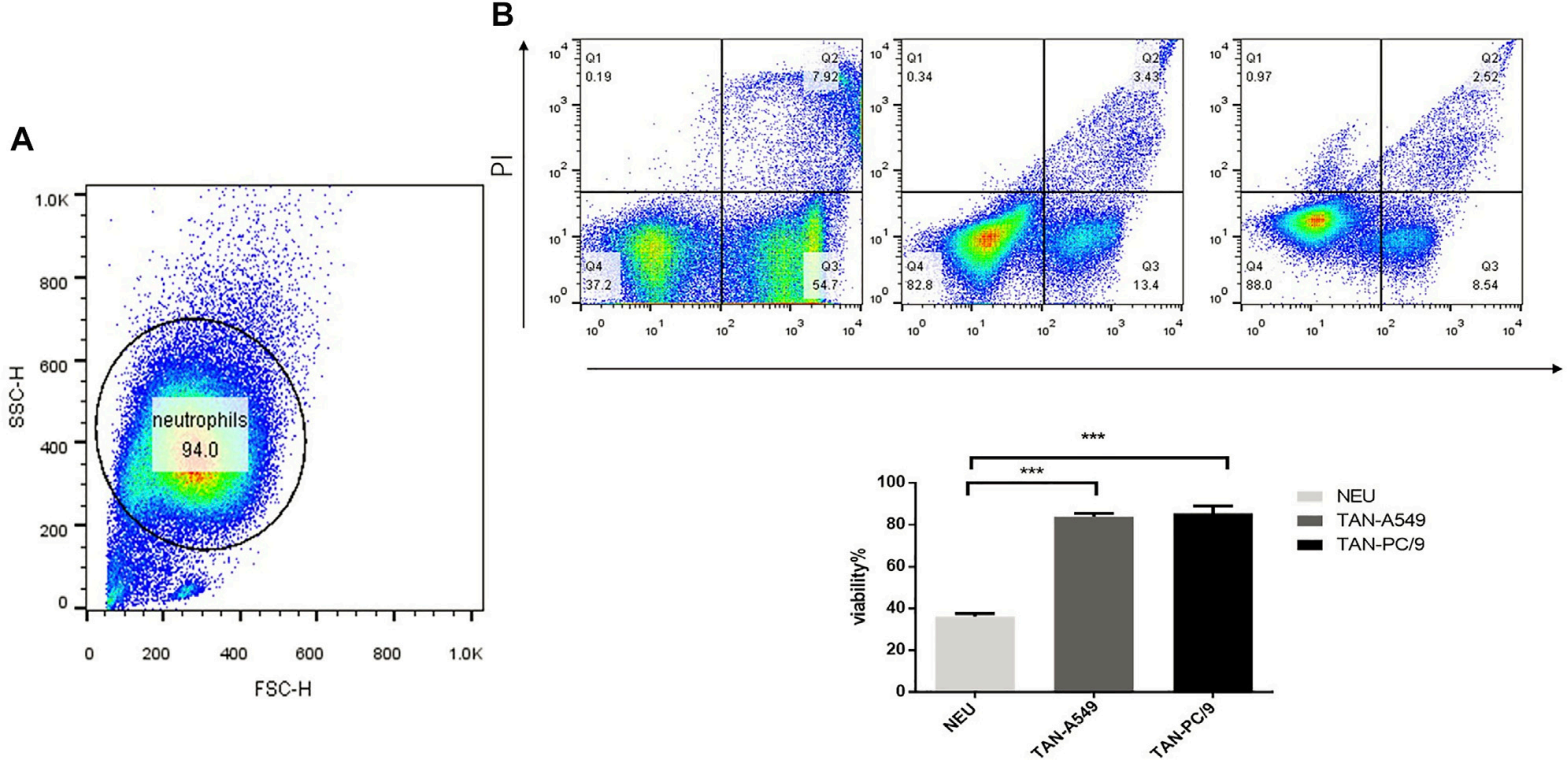
Supernatant of cultured lung adenocarcinoma cells prolonged the lifespan of neutrophils. **(A)** The purity of neutrophils was analyzed by flow cytometry. **(B)** The viability of neutrophils, neutrophils treated with the supernatant of cultured lung adenocarcinoma cells PC-9 and A549 respectively, was verified using flow cytometry.

The purity of neutrophils isolated from peripheral blood of healthy adults is 94.0% (Figure 2A). The supernatant of cultured A549 and PC-9 lung adenocarcinoma cell lines was used to stimulate neutrophils isolated from the peripheral blood of healthy adults for 10 h respectively. Compared with unstimulated neutrophils, a significant increase was observed in the lifespan of neutrophils treated with supernatant of cultured PC-9 (87.3% v. s 38.5%) and A549 (82.0% v. s.38.5%) cell lines (Figure 2B).

### The Migration and Invasiveness of Lung Adenocarcinoma Cells Were Promoted by TANs via Notch3

**FIGURE 3:**
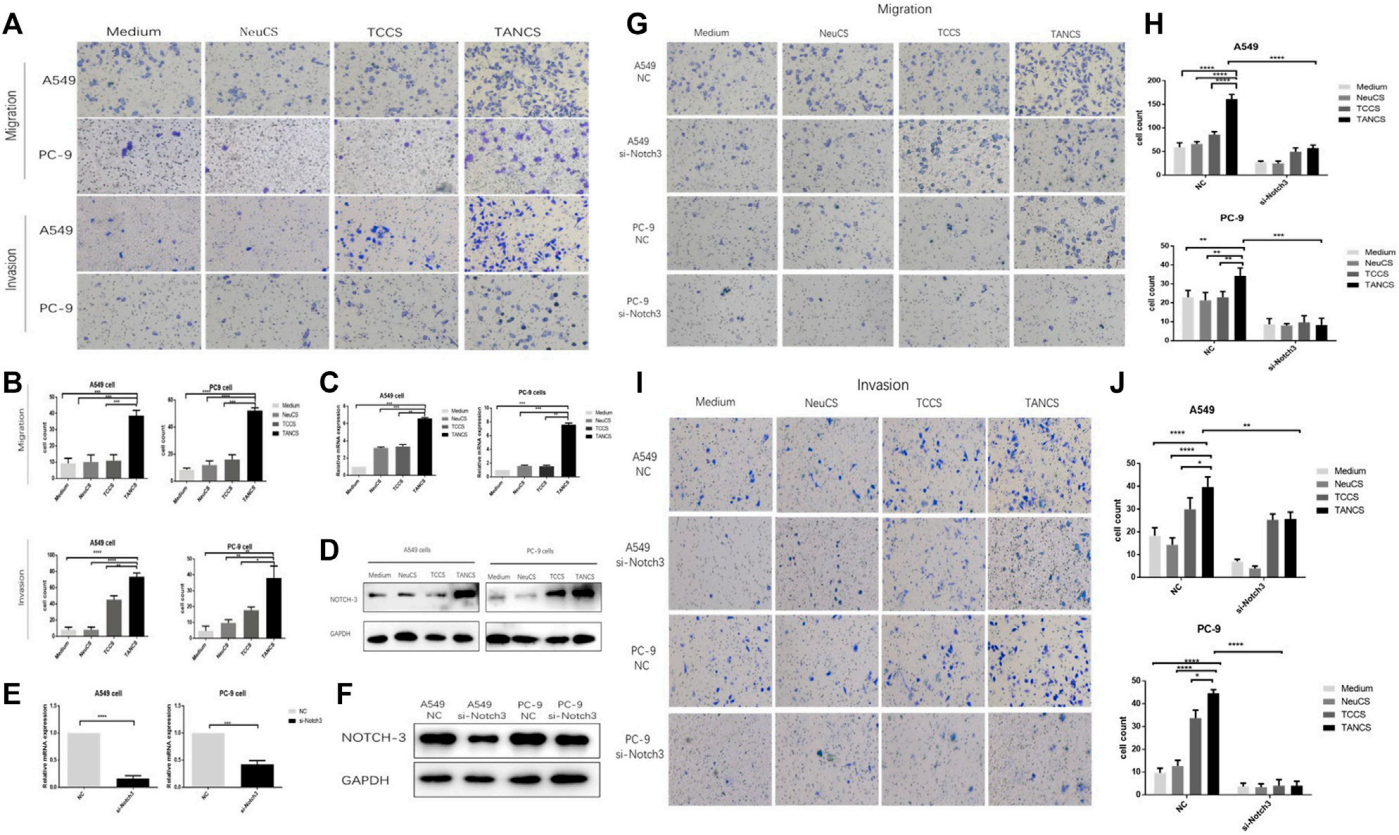
TANs promoted the migration and invasion of lung adenocarcinoma cells via Notch3. **(A,B)** A549 and PC-9 cells were stimulated with Medium, NeuCS, TCCS, and TANCS, respectively, then the migration and invasion of cells were observed. The expression of Notch3 in A549 and PC-9 cells stimulated with Medium, NeuCS, TCCS, and TANCS, respectively was detected by PCR assay **(C)** and Western blot assay **(D)**. The knockdown effect of specific siRNA (si-Notch3) in A549 and PC-9 cells was verified at both the mRNA **(E)**, by qRT-PCR) and protein levels **(F)**, by western blot). **(G–J)** A549-NC, A549-si-Notch3, PC9-NC, and PC9-si-Notch3 cells were stimulated by Medium, NeuCS, TCCS, TANCS, respectively, then the migration **(G–H)** and invasion **(I-J)** of the cells were observed.

Transwell assays were performed to investigate the effect of TANs on migration and invasiveness of lung adenocarcinoma cells. Compared with lung adenocarcinoma cells that were stimulated by medium, NeuCS, TCCS, the migration and invasion of lung adenocarcinoma cells stimulated by TANCS were significantly higher (Figures 3A,B). The same result was observed on the wound healing assay (Supplementary Figure S1).

Notch3 has been widely reported to be associated with cancer development. Therefore, whether Notch3 is involved in the migration and invasion of lung adenocarcinoma cells was explored. Our results showed a significant increase in the expression of Notch3 in lung adenocarcinoma cells treated with TANCS than that treated with medium, NeuCS, and TCCS respectively (Figures 3C,D). However, after knocking down of Notch3, the migration and invasion of lung adenocarcinoma cells stimulated with TANCS were not enhanced (Figures 3E–J and Supplementary Figure S2). The results suggested that TANs promoted the migration and invasion of lung adenocarcinoma cells via Notch3.

### Bioinformatics Analysis Showed That TANs and Notch3 Were Associated With Poor Prognosis

**FIGURE 4:**
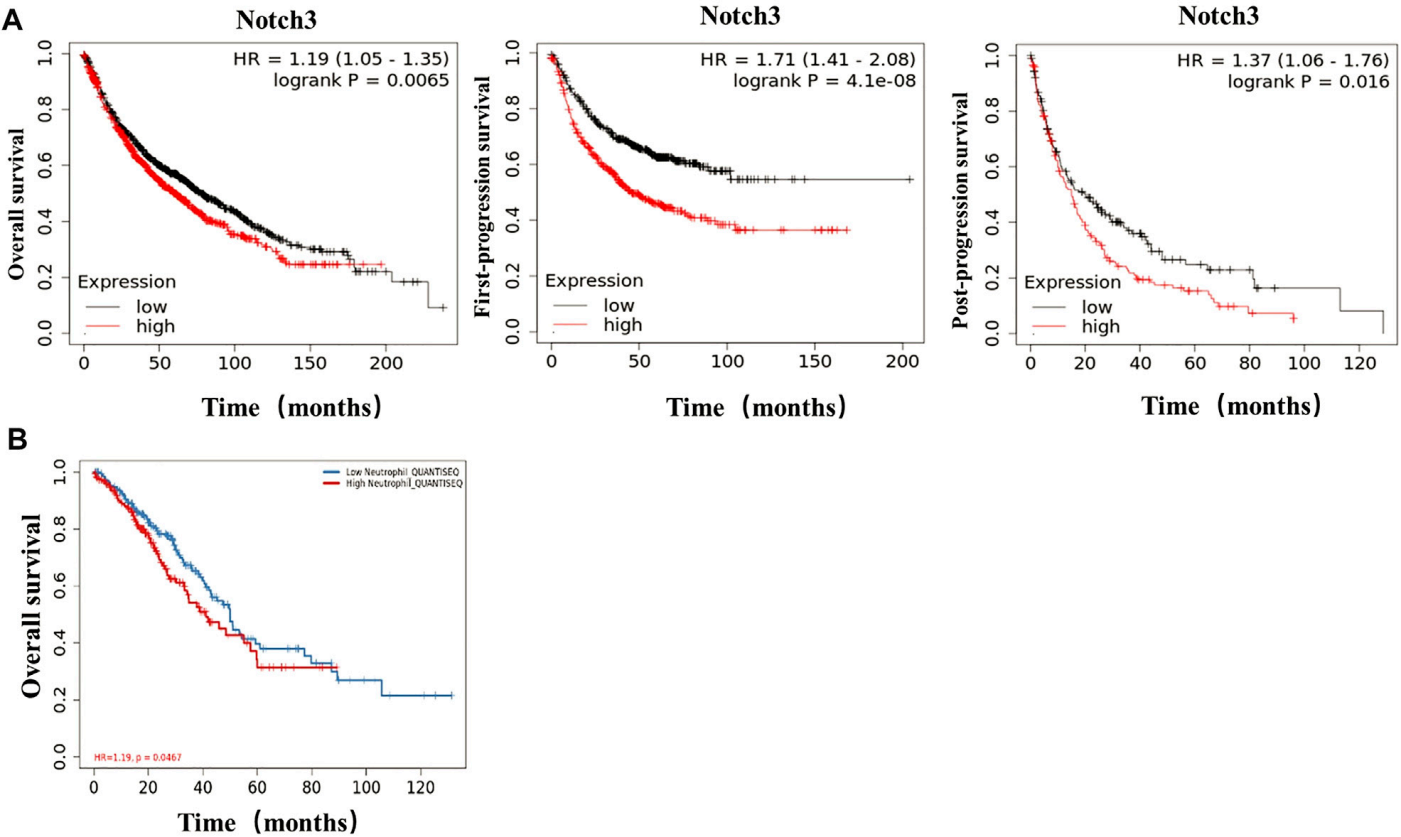
In lung adenocarcinoma, TANs and Notch3 correlate with poor prognosis. **(A)** The correlation of Notch3 and OS, FPS, PPS in lung adenocarcinoma was analyzed by Bioinformatics data. **(B)** The correlation of TANs and OS in lung adenocarcinoma was analyzed by Bioinformatics data.

The results from the Kaplan-Meier plotter showed that the expression of Notch3 correlated negatively with OS (overall survival), FPS (first-progression, survival), and PPS (post-progression survival) of patients (Figure 4A). From the TIMER (http://cistrome.dfci.harvard \.edu/TIMER/, Figure 4B) analysis, the infiltration of TANs was found to correlate negatively to the OS of patients. Our findings indicated that both TANs and Notch3 were associated with poor prognostic outcomes in patients.

## Discussion

Neutrophils, the most abundant immune cells in peripheral blood (Hidalgo et al., 2019), act as the body’s first line of defense against infection and respond to diverse inflammatory cues, including cancer (Shaul and Fridlender, 2019). Recently, the neutrophil-to-lymphocyte ratio in peripheral blood (pNLR) has been used as a significant prognostic factor for survival in many cancer types, including gastric cancer, melanoma, and lung cancer (Capone et al., 2018; Zhang et al., 2018; Sebastian et al., 2020). Although several studies have indicated that pNLR is associated with poor prognosis of cancer patients, the detection of pNLR can be influenced by various factors, including infection, medication, invasive surgery (Mouchemore et al., 2018).

In recent years, neutrophils infiltrated into tumors have gained attention as suitable biomarkers due to their ability to predict the progression of several cancer types. In gastric cancer, TANs are negatively correlated to the DFS of patients (Cong et al., 2020). In pancreatic neuroendocrine tumors, TANs have been shown to correlate negatively to the PFS and OS of patients (Zhang et al., 2020). Herein, we found the high density of TANs to be associated with poor prognostic outcomes in both immunohistochemical and Bioinformatics analysis. Our *in vitro* experiments revealed the abilities of TANs to promote the migration and invasion of A549 and PC-9 cells, which is consistent with the results found in other studies (Cong et al., 2020). Taken together, our findings suggest that the infiltration of TANs in lung adenocarcinoma can be further evaluated to predict the metastasis of tumor cells.

Several mechanisms regulating the effect of TANs on the metastasis of tumor cells have been reported. In gastric cancer, TANs promoted the migration and invasion of tumor cells by secreting IL-17a (Li et al., 2019). In colon cancer, TANs promoted the metastasis of tumor cells through the CCL15-CCR2 axis (Yamamoto et al., 2017). In our previous study, breast cancer cells induced neutrophil extracellular traps (NETs) by secreting IL-8, and these NETs promoted the migration of tumor cells (Cai et al., 2020). However, the tumor-promoting mechanisms of TANs with particular emphasis on lung cancer cell behavior are unclear.

According to numerous reports, notch-3 is associated with the development of cancer, including lung cancer (Aburjania et al., 2018). Ye and her co-workers discovered that Notch3 was associated with lymph node metastasis and poor prognosis (Ye et al., 2013). Therefore, we speculated that if TANs promoted the migration and invasion of lung cancer via Notch3. To verify this, we detected the expression of Notch3 in lung cancer by immunohistochemical staining and analyzed its correlation with TANs. We found Notch3 to be associated with poor prognosis and correlated positively with the infiltration of TANs. Results from our *in vitro* experiments demonstrated that TANs increased the expression of Notch3 in lung cancer cells. We further noted inhibition of the migration and invasion of lung cancer cells mediated by TANs after the knockdown of Notch3. Our findings suggest an interaction between Notch3 and neutrophils in the lung cancer tumor microenvironment. This is consistent with the results of Yang et al., who found TANs to promote the progression of ovarian cancer cells via the Notch3 pathway (Yang et al., 2020).

Collectively, our study indicated that lung adenocarcinoma cells promote self-invasion and self-migration by activating neutrophils to upregulate the Notch3 expression of cancer cells. Our study has given new insights into the pathogenesis of lung cancer. Targeting TANs with Notch3 could be a new diagnostic and therapeutic strategy for treating lung cancer.

## Data Availability

The raw data supporting the conclusions of this article will be made available by the authors, without undue reservation.
