# Supplementary figures and images for: Lung Adenocarcinoma Cells Promote Self-Migration and Self-Invasion by Activating Neutrophils to Upregulate Notch3 Expression of Cancer Cells
Source: Front Mol Biosci. 2022 Jan 18;8:762729. doi: 10.3389/fmolb.2021.762729 (PMC8804382; doi:10.3389/fmolb.2021.762729)

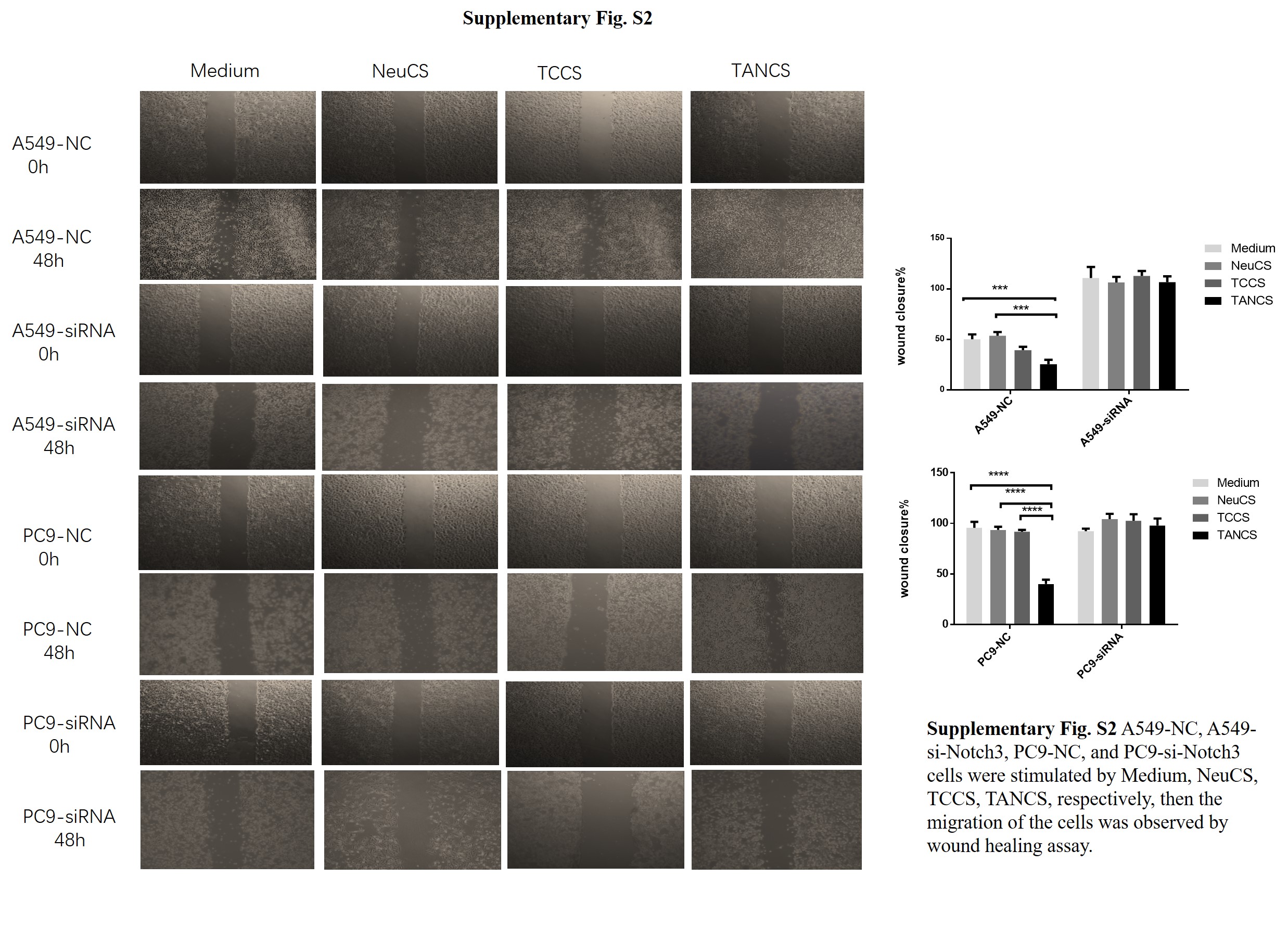

Supplement: Supplementary file 1 [file Image2.jpg]

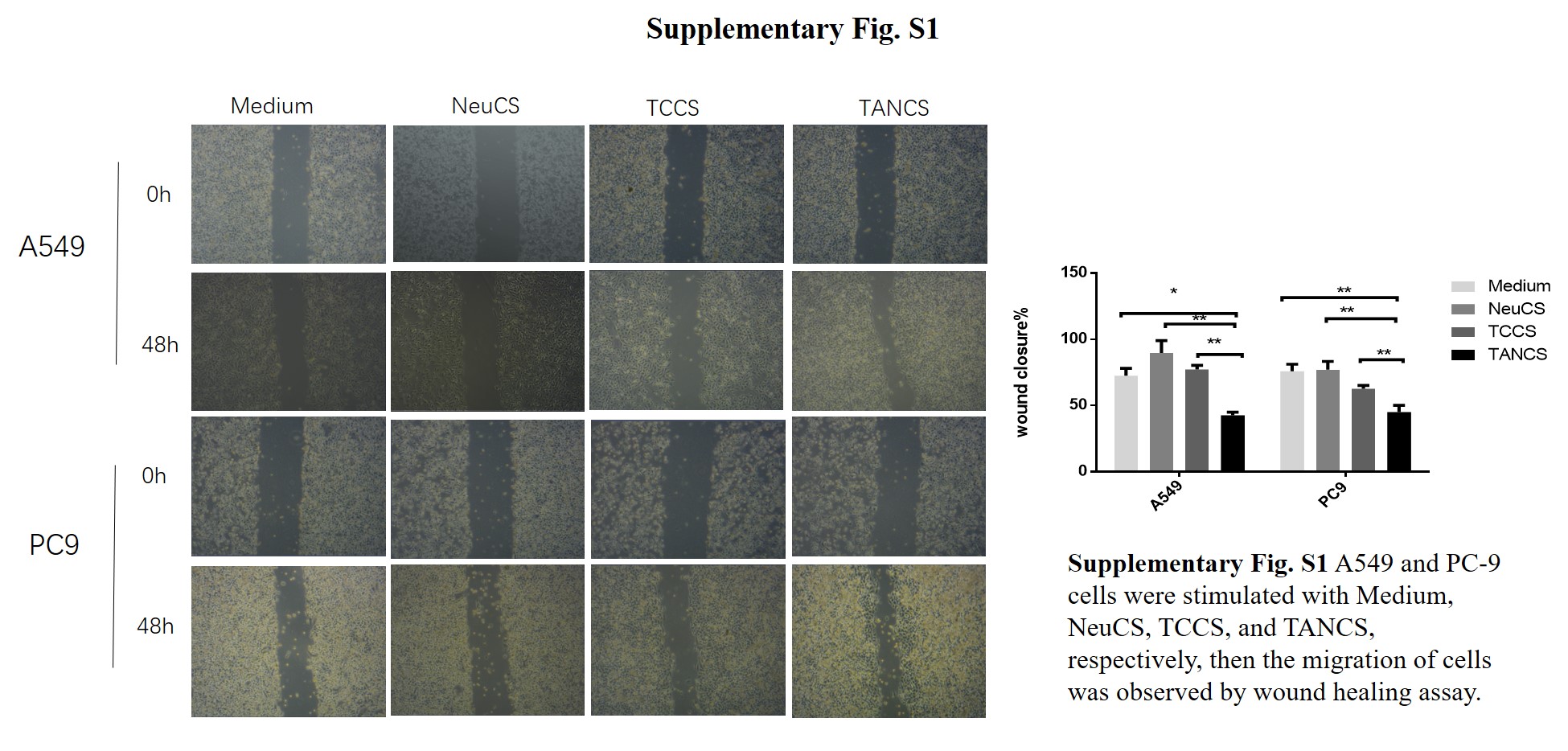

Supplement: Supplementary file 3 [file Image1.jpg]
