# Supplementary material for: Lung Adenocarcinoma Cells Promote Self-Migration and Self-Invasion by Activating Neutrophils to Upregulate Notch3 Expression of Cancer Cells
Source: Front Mol Biosci. 2022 Jan 18;8:762729. doi: 10.3389/fmolb.2021.762729 (PMC8804382; doi:10.3389/fmolb.2021.762729)
Supplement: Supplementary file 2 [file Table1.pdf]

Supplementary Table 1

Parameter	n	TANs		P value	Notch3		P value
		low	high		-	+	
Gender				0.5465			0.2922
Male	46	22	24		27	19	
Female	54	28	26		27	27	
Age				0.4193			0.3235
＜60	57	26	31		28	29	
⩾60	43	24	19		26	17	
Tumor ststus				0.7288			0.5654
T1	46	25	21		27	19	
T2	42	19	23		22	20	
T3	6	3	3		2	4	
T4	6	3	3		3	3	
TNM stage				0.0348			0.6001
I+II	66	38	28		36	30	
III+IV	34	12	22		18	16	
Differentiation				0.0473			0.0032
Moderate+well	71	40	31		45	26	
Poor	29	10	19		9	20	
Pathological type				0.0133			0.0058
Lepidic predominant	20	13	7		10	10	
Acinar predominant	46	26	20		32	14	
Papillary predominant	9	6	3		6	3	
Micropapollary predominant	6	1	5		2	4	
Solid predominant	19	4	15		4	15	
Lymph node Metastasis				0.0938			0.3387
+	59	30	29		34	25	
-	41	20	21		20	21	

Infiltrating of TANs and expression of Notch3 in lung adenocarcinoma and their clinicopathological significance
